# Hématome spontané du rein

**DOI:** 10.11604/pamj.2017.26.75.10634

**Published:** 2017-02-20

**Authors:** Adil Kallat, Hani Abousaleh

**Affiliations:** 1 Service d’Urologie A, Hôpital Ibn Sina, CHU Rabat, Maroc

**Keywords:** Hématome, spontané, rein, Hematoma, spontaneous, kidney

## Image en médecine

Il s’agit d’un patient de 54 ayant comme antécédents une appendicectomie il y a 6 ans et qui présente des lombalgies gauches évoluant depuis 3 mois aggravées 3 jours avant son admission aux urgences. Par ailleurs il n’y avait pas de notion d’hématurie ou d’émission de calculs ni de traumatisme lombaire ou abdominal. L’examen à l’admission retrouvait un patient en assez bon état général, conjonctives légèrement décolorées, stable sur le plan hémodynamique. Le bilan biologique retrouvait une hémoglobine à 7.7g/dl, le reste du bilan était sans particularité. L’uroscanner réalisé mettait en évidence la présence d’une volumineuse collection péri-rénale gauche à prédominance postérieure, hétérogène, renfermant des zones spontanément hyperdenses (A). Le patient a été transfusé de deux culots globulaires. Vu la stabilité hémodynamique, on avait décidé ce faire une simple surveillance avec l’instauration d’un traitement antalgique et antibiotique pour éviter la surinfection de l’hématome. L’uroscanner de contrôle a été réalisé un mois plutard ayant objectivé une nette régression de l’hématome (B).

**Figure 1:**
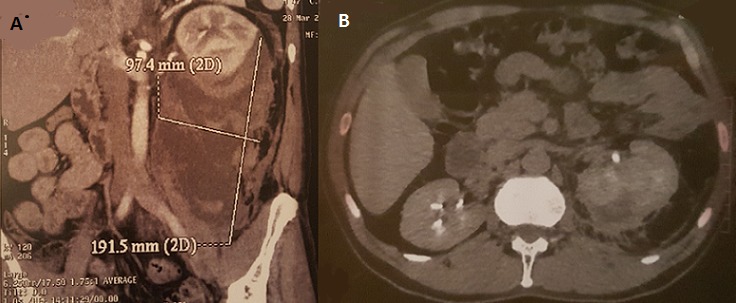
A) scanner abdominal montrant un gros hématome péri rénal gauche; B) scanner abdominal montrant une nette régression de l’hématome péri rénal gauche

